# Environmentalism in the EU-28 context: the impact of governance quality on environmental energy efficiency

**DOI:** 10.1007/s11356-019-06600-1

**Published:** 2019-11-19

**Authors:** Nicholas Apergis, Claudia Garćıa

**Affiliations:** 1 University of Derby, Derby, UK; 2 University of Granada, Granada, Spain

**Keywords:** Environmental policies, Governance quality, Energy efficiency, EU-28, Panel data, Q4, Q50, O52, O57, C51, C52

## Abstract

Environmental policies are a significant cornerstone of a developed economy, but the question that arises is whether such policies lead to a sustainable growth path. It is clear that the energy sector plays a pivotal role in environmental policies, and although the current literature has focused on examining the link between energy consumption and economic growth through an abundance of studies, it does not explicitly consider the role of institutional or governance quality variables in the process. Both globalization and democracy are important drivers of sustainability, while environmentalism is essential for the objective of gaining a “better world.” Governance quality is expected to be the key, not only for economic purposes but also for the efficiency of environmental policies. To that end, the analysis in this paper explores the link between governance quality and energy efficiency for the EU-28 countries, spanning the period 1995 to 2014. The findings document that there is a nexus between energy efficiency and income they move together: the most efficient countries are in the group with higher GDP per capita. Furthermore, the results show that governance quality is an important driver of energy efficiency and, hence, of environmental policies.

## Introduction

Nowadays, environmental policies are a significant cornerstone of a developed economy. As Jebli et al. (2016) illustrate the big problem is that the consumption of non-renewable energy (i.e., oil, coal, and natural gas) not only increases economic growth but also increases carbon dioxide emissions. These emissions are considered as the main cause of global warming. Therefore, it is necessary to increase the energy efficiency and to find substitutable energy to the fossil one, such as renewable energy. Similarly, Lloyd (2017) notes that economic growth is associated with greater levels of energy consumption, but that, in turn, implies that there is a conflict between growth and the desire to reduce emissions. Bano et al. (2018) states the knowledge-based economies, advanced technology, and globalization motivate everyone to find the most appropriate way to maintain the competitive advantage, as well as carbon emissions mitigation.

In contrast, environmental degradation occurs because global economies plan to basically generate business and employment opportunities, rather than to support the environmental quality at the first stages of development (Apergis et al. 2018). In this work, the authors conclude that by using clean/renewable technologies, it can substantially reduce emissions of climate change pollutants; therefore, they recommend economies to increment energy consumption coming from renewable sources. But one challenge appears: Barros et al. (2013) and Apergis and Payne (2014) note that it is critical for the development of a sustainable energy consumption mix for policymakers to understand that policy initiatives must focus on cost-effective renewable energy sources, as well as on technologies that can effectively compete with fossil fuel–based energy sources.

From the above, it is getting clear that energy sources play a pivotal role in environmental policies, and following certain authors, such as Aklin et al. (2013) or Bernauer and Koubi (2009), who defend that public opinion may be a powerful determinant of environmental policies in a developed country would be substantially interesting to explore the relationship between certain public sector variables and the energy sector, from an environmental point of view. As Blühdorn and Welsh (2007) argue, we are in a new era and eco-politics needs a new environmental sociology. The “Theoretical framework” section will further develop this idea. Basically, the aim of this study is to consider two important issues: first, whether the European Union is energetic-sustainable efficient, and second, whether the quality of governments can impact this efficiency. To the best of our knowledge, this work is the first to analyze the impact of governance indicators on sustainable efficiency in a country context. The findings document that the government quality variables can improve both environmental quality and energy efficiency, while they are equally important in explaining any improvements in the environmental quality.

The remaining of the manuscript is organized as follows: the “Theoretical framework” section further studies the theoretical framework and previous research in this field. The “Data and variables” section describes the variables and data used, while the “Methodological analysis and final results” section provides the methodological analysis, including the baseline results, as well as certain robustness checks. The “Empirical analysis” section provides the empirical analysis and discussion. Finally, the “Discussion and conclusion” section concludes and provides certain implications.

## Theoretical framework

Based on the above discussion, it emerges the concept of the environmental risk, defined as the probability of damages to any community, due to the vulnerability of its environmental components exposed to human activities. The onset of this risk implies that changes in the economic structure, as well as in the associated policies, should be carried out. For Greenpeace^1^ or NASA^2^, the solution comes from the energy sector with the use of renewable energy sources. In the European Union (EU) context, the Europe 2020 strategy is a policy for years 2010–2020 that emphasizes smart, sustainable, and inclusive growth as a way to overcome any structural weaknesses in the European economy, to improve its competitiveness and productivity, and to underpin a sustainable social market economy^3^. Some of the principal goals of the Europe 2020 strategy are the climate change and energy targets whose principal actions can be summarized into two major ways: diminishing the emissions to the atmosphere and increasing the energy efficiency of the countries. More specifically, there are three targets (for 2020) into this headline: (i) diminishing the greenhouse gas (GHG) emissions to 20% than 1990 levels, (ii) increasing by 20% of renewable energies use, and (iii) increasing by 20% total energy efficiency. For 2030, the targets expand: reduction by 40% of greenhouse gas emissions, level of EU energy from renewables at least of 27%, increase energy efficiency by 27–30% and there has to be a level of 15% of electricity inter-connection between the EU members. Thus, EU energy policies have three main goals: the security of supply, competitiveness, and sustainability; Europe should become a sustainable, low-carbon, and environmental-friendly economy, while it will lead the way in renewable energy production and the fight against global warming. The EU has provided some information about environmental and energy target objectives^4^: GHGs should be reduced by 18% between 1990 and 2012; renewables’ share should reach 14.1% in 2012, up from 8.5% in 2005; and energy efficiency is expected to improve by 18–19% by 2020.

From the above, it is clear that the energy sector plays a pivotal role in environmental policies. It will be highly interesting to investigate whether the energy sector exposes high efficiency, from an environmental point of view. In this line, previous research has focused on investigating the relationship between energy consumption and economic growth and/or pollutant emissions (Ahmad et al. 2016; Apergis et al. 2010, 2018; Apergis and Payne 2014; Coondoo and Dinda 2002; Dinda 2004; Jebli et al. 2016; Omer 2008; Sadorsky 2009a, 2009b; Tugcu et al. 2012), among others. Liobikieǹe and Butkus 2018 assume that both energy efficiency and renewable energy consumption are the main drivers that could resolve the problem of climate change. However, it can be considered that both factors are, in fact, similar. A large number of studies have already confirmed this bidirectional causality (Liobikieǹe and Butkus 2018; Shahbaz et al. 2013; Wang et al. 2015). Mikayilov et al. (2018) note that a suitable environmental policy to reduce total CO2 emissions without harming economic growth is to improve energy efficiency, which can be obtained by increasing optimal infrastructure investment and employing energy conservative policies to avoid unnecessary use of energy. Put differently, using less energy intensive technologies, minimizing the loss of power during distribution and transmission processes, and employing different tariff mechanisms to control energy use are some applicable policies that are capable to increase energy efficiency, while Tajudeen et al. (2018) observe that energy efficiency improvements are most cost-effective and most readily scalable options to support sustainable growth.

Apart from energy sources, a different strand of research has incorporated many factors in sustainable growth, such as innovations, population, financial variables, and trade (Anderson and Mizak 2006; Begum et al. 2015; Komal and Abbas 2015; Nasreen and Anwar 2014; Sohag et al. 2015; Wang et al. 2016a, 2016b, among others); nevertheless, there are also other relevant factors that could also impact the environmental enhancement. As we have already mentioned in the “Introduction” section, public opinion may be a powerful determinant of environmental policies in a developed country (Aklin et al. 2013; Bernauer and Koubi 2009). Furthermore, Salahodjaev (2016) emphasizes globalization, democracy, and the institutional environment as potential drivers of environmental sustainability. More specifically, institutional characteristics could be also important drivers in this field (Barbier 1997; Bhattarai and Hammig 2001; Deacon 1994; Norton 1998; Samuelson 1976). Bhattarai and Hammig (2004) test that economic development is accompanied by the deterioration of environmental quality following an inverted U-shape (i.e., the Environmental Kuznets Curve hypothesis), while they assume that increases in income are associated with improvements in socio-political institutions, and concluding that the quality of governance is a critical determinant of environmental degradation (measured by deforestation). In contrast, economic freedom is a concept which can have differing relations with certain economic factors, such as income inequality, economic growth, democracy, and human development. Eastin and Prakash (2013) explore the relationship between economic growth and an important component of economic freedom, that of gender inequality. Their findings illustrate that the relationship between these two variables is very complex and has to be taken explicitly into account by policymakers. Li et al. (1998) find that there is solid evidence for a close association between income inequality and democracy: *the rich are able to exercise sufficient control over economic policy at least to maintain their wealth [...], again reinforcing the tendency for unequal distributions of income*. Apergis and Cooray (2017) and Apergis et al. (2014) investigate the nexus between income inequality and the economic freedom index and they document that there is an inverse relationship between them. Farzin and Bond 2006 put forward an important issue: As a general rule, political and civil liberties are instrumentally powerful in protecting the environmental resource base, at least when compared with the absence of such liberties in countries run by authoritarian regimes. This observation raises several important questions: How does pubic environmental policy influence the relationship between per capita income and pollution?

Institutional variables have been extensively considered in the economic and political structure relationships (Berggren 2003; Chortareas et al. 2011, 2012, 2013, 2016; De Haan et al. 2006; Demirgüç-Kunt et al. 2003; Gwartney et al. 2006; Lin et al. 2016; Pitlik 2002), among others. Because environmental quality may be considered as a public good, environmental policies are at least partly influenced by the society’s preferences for environmental quality, while they reflect the degree of democratization, as well as the quality of political institutions (Farzin and Bond 2006). Rivera-Batiz (2002) expresses that one of the major determinants of environmental policy is the socio-political regime of a particular country (i.e., the quality of governance). Furthermore, Magnani (2000) argues that well-defined property rights, democratic voting systems, and the respect of human rights can generate synergies that lead to increased levels and the efficacy of environmental policies. In addition, as Gnonlonfin et al. (2017) or Dinda (2004) highlight, the presence of the relation between income and pollution depends on certain traditional factors, such as technology, trade, etc., as well as on the role of the State, institutions and regulation policies. Tajudeen et al. 2018 conclude that non-economic factors (such as, the characteristics of consumers in relevance to their preferences or their environmental awareness) have a significant influence on energy demand and, hence, on CO2 emissions, while they document that these factors have a direct influence on environmental damage. Hence, it is clear that energy use affects sustainability across all its fundamental components, society, environment, and economy (Tronchin et al. (2018)). Additionally, the current literature hypothesizes that the membership in international organizations tends to improve both the environmental performance and the likelihood of joining international environmental treaties (Shahbaz et al. 2018; and reference therein). Therefore, our study focuses on the EU, which may be considered one of the international organizations that is more aware of sound environmental practices.

Finally, effective policies would have a positive effect on environmental conservation, especially, for countries that exhibit carbon-emitting attitudes (Tajudeen et al. 2018), like the EU-28 club. It is clear that one of the keys in environmental improvement is the energy sector, and more specifically, its energy efficiency. The main goal of this paper is to clarify whether institutional variables have any influence on energy efficiency: the major question this study poses is whether certain characteristics of institutions, as corruption and/or rule of law play a pivotal role in environmental damage. We could consider that such variables are substantially relevant, especially, in the energy sector, which has been traditionally a very concentrated sector and with a high level of intervention from the governments’ side. EU energy policies have three main goals: the security of supply, competitiveness and sustainability; all these ensure a secure, affordable and climate-friendly energy environment for all EU citizens and businesses: Europe should become a sustainable, low-carbon and environmentally friendly place, and it should lead the way in renewable energy production, as well as the fight against global warming^5^. In the EU context, it is clear that the energy sector plays a key role in climate change and environmental damage, and furthermore, the governments have to ensure that their energy objectives are fully met in an efficient and coherent way. To that end, it is highly important to observe whether this sector is efficient or it has to improve its efficiency, while we simultaneously need to explore whether the individual qualitative characteristics of the member governments can impact its efficiency.

To the best of our knowledge, this work is the first to analyse the effect of governance quality on environmental degradation, particularly, on environ- mental and energy efficiency in the context of EU. The analysis covers all members of the EU-28, except Malta, spanning the period 2002 to 2014.

The analysis will investigate the validity of the following two principal hypotheses:

Hypothesis 1: Can the energy sector improve its “sustainable” efficiency? and Hypothesis 2: Is governance quality important in explaining the efficiency in the energy sector?

The results document that the government quality variables are capable of improving both environmental quality and energy efficiency, while they are important in explaining any improvements in the environment.

## Data and variables

### The dependent variable: the DEA and energy efficiency sustainable index

The first part of the methodology measures the energy efficiency sustainable index. To that end, it employs the methodology of data envelopment analysis (DEA) approach, proposed by Charnes et al. (1978). It is a well-established non-parametric frontier approach that assesses and measures the relative efficiency of a set of comparable entities (called decision making units or DMUs) featured with multiple factors grouped into two categories: inputs and outputs. Classical DEA models rely on the assumption that inputs have to be minimized and outputs have to be maximized (Vencheh et al. 2005). Thus, in the standard DEA model, decreases in outputs are not allowed; only inputs are allowed to decrease (similarly, increases in inputs are not allowed, while only outputs are allowed to increase) (Seiford and Zhu 2002), but the production process could generate also undesirable outputs (pollutants).

There are several approaches for incorporating undesirable outputs in DEA modeling approach. These models can also be classified into two groups: the ones that take an indirect perspective and the ones that take a direct approach. As Scheel (2001) argues, indirect approaches transform the values of the undesirable outputs through a monotone decreasing function, such that the transformed data can be included as desirable outputs in the technology set; direct approaches can use the original output data set, but modify the assumptions about the structure of the technology set in order to treat the undesirable outputs appropriately. As Scheel (2001) remarks, the indirect approaches assume that the transformed data have their own meaning; for instance, if we transform the undesirable output mortality rate, then we can study the desirable output survival rate. In contrast, the direct approach employs the original output set, but it changes the assumptions adopted. The direct approach, suggested by Färe et al. (1989), replaces the strong disposability of outputs by the assumption that outputs are weakly disposable, while only the sub-vector of desirable outputs is strongly disposable. The direct approach is preferable implying that it is not necessary for researchers to make any changes to the main dataset, while it is not necessary to reinterpret the results obtained in terms of the “new” variables (e.g., mortality and survival rates). The analysis in this work makes use of DEA method, focusing on the direct approach, to calculate the energy efficiency sustainable index across the EU-28 members. It considers one of the models developed by Zhou and Ang 2008, who measure the energy efficiency performances of 21 OECD countries. The reason of using this particular model is that the analysis focuses on the technical efficiency of energy consumption. The technical efficiency is defined as the ability of a DMU to obtain maximal outputs (or minimal inputs) from a given set of inputs (or a given set of outputs (Robaina-Alves et al. 2015; Moutinho et al. 2017). The principal advantage of using the DEA method is its flexibility to incorporate factors incomparable a priori (both inputs and outputs) that makes the results easily interpretable. As Balk et al. 2017 illustrate, the DEA method searches for the most favorable weight when evaluating a production unit, by constructing a virtual aggregate input to output productivity ratio, each constructed as a linear combination of observed values.

Assume that the set of DMUs consists of DMU_*k*_, *k* = 1, ⋯*K*. Let **x**_*nk*_ = (**x**_1*k*_,  **x**_2*k*_, ⋯, **x**_*Nk*_), **e**_*lk*_ = (**e**_1*k*_,  **e**_2*k*_, ⋯, **e**_*Lk*_), **y**_*mk*_ = (**y**_1*k*_,  **y**_2*k*_, ⋯, **y**_*Mk*_) and **u**_*jk*_ = (**u**_1*k*_,  **u**_2*k*_, ⋯, **u**_*Jk*_) are the vectors of non-energy inputs, energy inputs, desirable outputs, and undesirable outputs, respectively. The efficiency score of DMU_*i*_ can be obtained by solving model (1) below.

1 $$ {\displaystyle \begin{array}{c}\min {\theta}_i\kern3.25em r.t.\\ {}\sum \limits_{k=1}^K{z}_k{\mathbf{x}}_{nk}\le {\mathbf{x}}_{ni},\kern0.5em n=1,\cdots, N\\ {}\begin{array}{c}\sum \limits_{k=1}^K{z}_k{\mathbf{e}}_{lk}\le {\theta}_i{\mathbf{e}}_{li},\kern0.5em l=1,\cdots, L\\ {}\sum \limits_{k=1}^K{z}_k{\mathbf{y}}_{nk}\ge {\mathbf{y}}_{mi},\kern0.5em m=1,\cdots, M\\ {}\begin{array}{c}\sum \limits_{k=1}^K{z}_k{\mathbf{u}}_{nk}={\mathbf{u}}_{ji},\kern0.5em j=1,\cdots, J\\ {}{z}_k\ge 0,\kern0.5em k=1,2,\cdots, K\end{array}\end{array}\end{array}} $$

“It can be seen that [model (1)] attempts to proportionally contract the amounts of energy inputs as much as possible for a given level of non-energy inputs, desirable and undesirable outputs. It provides an aggregated and standardized index for measuring energy efficiency performance” (Zhou and Ang (2008); pp. 2913). The higher value is, the better situation for each DMU is. The maximum possible value is one, which implies that the DMU is relatively efficient, regarding the rest of DMUs. In contrast, if the value of the index is zero (the minimum possible value), it implies that the DMU is relatively inefficient.

It is important to remark that the DEA approach has certain limitations, despite the attractiveness of its application. More specifically, the weight flexibility, previously explained, may lead to unreasonable results, inconsistent with any prior knowledge of the production process (Balk et al. (2017)); in that sense, the results must be analyzed carefully and compared with the theoretical framework and previous research. Our case does not encounter this problem and the results are consistent (“Methodological analysis and final results” and “Empirical analysis” sections). In addition, it does not allow the comparison of different results “externally”; the results of the analysis can be only compared “internally” in a sense that we are not able to compare the findings with any other dataset, which would offer other different scores. DEA measures the relative efficiency of DMUs that perform similar type of functions and have identical goals and objectives; for instance, if we analyze a particular group of countries, we may not compare these results with any other groups, even in the case we add only an additional country. Apart from this little inconvenience, the use of DEA provides the flexibility of the application: it is not necessary to explicitly specify a priori a production function that explains how the inputs and outputs of the production units are linked to each other (Cecchini et al. (2018)). Furthermore, DEA has emerged in recent years as a highly sophisticated method to assess efficiency measures, and particularly, environmental efficiency across countries and economic sectors (Robaina-Alves et al. (2015)).

Once the methodology is clarified, this part defines the index. The data used for obtaining the dependent variable include, as we have anticipated, non-energy and energy inputs, as well as two types of outputs, i.e., desirable and undesirable, to measure the sustainable efficiency. In the case of energy inputs, we have two groups of variables: first, we get the efficiency scores using group 1 (with only one energy input: energy use), and then using group 2 (with two energy inputs: energy consumption distinguishing between fossil and non- fossil energies). The final dependent variable (called E in following sections) is the average of these two energy efficiency scores. The reason of building the dependent variable as above is to balance the energy efficiency results for dealing with the weight flexibility problem previously mentioned. Next, the factors are measured as follows^6^:

- Non-energy inputs:
  - Labor force (total, people ages 15 and older).
  - Gross capital formation (% of GDP).
- Energy inputs:
  - Group 1: Energy use (kg of oil equivalent per capita).
  - Group 2:
- Fossil fuel energy consumption (% of total).
- Renewable energy consumption (% of total).
- Desirable output: GDP per capita, PPP (constant 2011 international $).
- Undesirable output: CO_2_ emissions (kt).

**Fig. 1:**
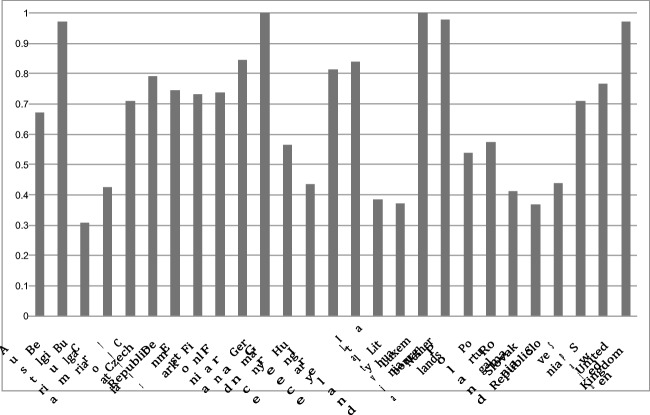
Average of energy efficiency scores for each member: EU-28 (excluding Malta), 1995–2014

**Fig. 2:**
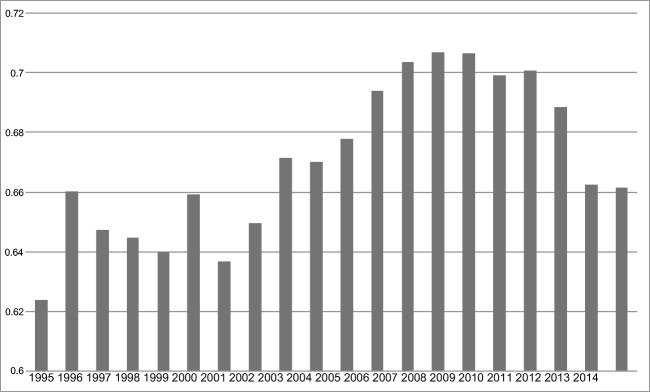
Average of energy efficiency scores for each year: EU-28 (excluding Malta), 1995–2014

The analysis will provide the results for the energy efficiency sustainable index (**E**) across the different members of EU-28 (excluding Malta) for each year, from 1995 to 2014 (last data available for CO_2_ emissions). Malta has been excluded due to certain data unavailability. The dependent variable, **E**, is called throughout the paper as sustainable or environmental energy efficiency because of the incorporation of the undesirable output, CO_2_ emissions, which allows to obtain the energy efficiency scores taking into account the ecological effect of the economy on the environment. The results are reported in Table 1. In addition, Figs. 1 and 2 display the average of each country and the average of each year for the total EU-28 (excluding Malta), respectively.

**Table 1:** Energy efficiency scores for EU-28 (excluding Malta), 1995–2014

	1995	1996	1997	1998	1999	2000	2001	2002	2003	2004	2005	2006	2007	2008	2009	2010	2011	2012	2013	2014
Austria	0.7117	0.7149	0.6987	0.6747	0.6701	0.6845	0.6467	0.667	0.6535	0.658	0.6598	0.6813	0.6768	0.6872	0.7047	0.6712	0.6653	0.6723	0.6235	0.6102
Belgium	1	1	1	1	1	1	1	1	1	1	1	1	0.9259	1	1	1	1	0.922	0.8331	0.7809
Bulgaria	0.2101	1	0.2205	0.2308	0.223	0.2287	0.2259	0.2432	0.2557	0.2609	0.2676	0.2757	0.2887	0.313	0.336	0.3278	0.3123	0.3223	0.3217	0.3057
Croatia	0.39	0.432	0.4187	0.4143	0.3937	0.4204	0.4062	0.4131	0.429	0.4122	0.418	0.4316	0.4345	0.4637	0.4528	0.4391	0.4369	0.4461	0.4165	0.4155
Cyprus	0.7389	0.6831	0.7565	0.8878	0.8313	0.8869	0.7092	0.6707	0.6717	0.6765	0.6778	0.675	0.6662	0.6725	0.6789	0.6706	0.665	0.6629	0.6487	0.6421
Czech Republic	0.6603	0.5845	0.7993	0.6124	0.5214	0.8296	0.7749	0.8624	0.8591	0.7423	0.9677	1	1	1	0.959	0.825	0.9495	0.7885	0.5341	0.5829
Denmark	0.7738	0.8109	0.7692	0.7309	0.7401	0.7578	0.717	0.7455	0.738	0.7696	0.7785	0.7499	0.7254	0.7401	0.7555	0.7184	0.7131	0.7521	0.6823	0.7099
Estonia	0.4903	0.5237	0.5733	0.4932	0.4597	0.4977	0.4999	0.564	0.6696	0.7035	0.7777	0.7346	0.9578	0.8877	0.7783	1	1	1	1	1
Finland	0.5864	0.6693	0.6952	0.6482	0.648	0.6079	0.63	0.7749	0.9276	0.9446	0.6847	1	0.8222	0.7756	0.7847	0.8595	0.8372	0.6748	0.6173	0.5764
France	0.823	0.81	0.8236	0.8301	0.8389	0.8428	0.833	0.8357	0.8473	0.843	0.849	0.8527	0.8595	0.8601	0.8641	0.8604	0.8643	0.863	0.8565	0.8484
Germany	1	1	1	1	1	1	1	1	1	1	1	1	1	1	1	1	1	1	1	1
Greece	0.5481	0.5737	0.5609	0.5577	0.5491	0.543	0.5404	0.552	0.5896	0.5756	0.6043	0.5893	0.6524	0.6406	0.6841	0.5794	0.5183	0.4887	0.4852	0.478
Hungary	0.3758	0.383	0.3855	0.4064	0.3989	0.4219	0.4096	0.4227	0.4442	0.4397	0.4385	0.4476	0.4502	0.4668	0.4716	0.4504	0.4546	0.4672	0.4643	0.4652
Ireland	0.8018	0.7398	0.9105	0.9674	0.9982	1	0.9242	0.7803	0.7637	0.7668	0.7745	0.7692	0.7934	0.7576	0.7542	0.7443	0.7491	0.7595	0.7537	0.7649
Italy	0.8417	0.8406	0.8396	0.8405	0.8389	0.839	0.837	0.8426	0.8519	0.8523	0.863	0.8467	0.9249	0.8391	0.838	0.8269	0.8265	0.8151	0.8026	0.7872
Latvia	0.2469	0.2714	0.2865	0.3082	0.3231	0.3481	0.3387	0.3655	0.3935	0.3874	0.4144	0.4418	0.4701	0.4824	0.4457	0.4025	0.4413	0.4578	0.4371	0.441
Lithuania	0.2525	0.2673	0.2827	0.2926	0.3011	0.3342	0.314	0.3302	0.3681	0.3593	0.3785	0.401	0.4174	0.4403	0.4254	0.4351	0.4424	0.4566	0.4587	0.4655
Luxembourg	1	1	1	1	1	1	1	1	1	1	1	1	1	1	1	1	1	1	1	1
Netherlands	1	1	0.9564	0.982	0.999	0.9429	0.8571	0.9107	0.9364	0.9946	1	0.9842	0.9771	1	1	1	1	1	1	1
Poland	0.4974	0.4959	0.5012	0.4911	0.4972	0.4966	0.4966	0.4985	0.5086	0.5226	0.5308	0.5435	0.5648	0.5633	0.5875	0.5869	0.6092	0.5939	0.5963	0.6172
Portugal	0.5908	0.6486	0.6244	0.611	0.5745	0.5887	0.576	0.558	0.5845	0.5428	0.5288	0.5468	0.5529	0.5745	0.576	0.5866	0.5709	0.5721	0.5298	0.5223
Romania	0.3174	0.3207	0.3158	0.327	0.3465	0.3484	0.356	0.361	0.3817	0.3986	0.4112	0.4246	0.4489	0.489	0.5123	0.4896	0.4773	0.4901	0.5103	0.5071
Slovak Republic	0.2744	0.2998	0.3038	0.3447	0.3179	0.3286	0.2965	0.3079	0.3341	0.3325	0.3438	0.3679	0.4035	0.4296	0.4411	0.4325	0.4432	0.4656	0.4404	0.4558
Slovenia	0.3923	0.4139	0.4048	0.417	0.4153	0.4328	0.4174	0.4302	0.4512	0.4305	0.436	0.4465	0.4643	0.4762	0.4803	0.4619	0.4596	0.4552	0.4283	0.441
Spain	0.6751	0.6976	0.6813	0.6896	0.6885	0.6893	0.6902	0.6963	0.718	0.7082	0.7245	0.7254	0.734	0.7399	0.7501	0.7376	0.7163	0.7037	0.6997	0.6925
Sweden	0.7532	0.7574	0.7591	0.7379	0.746	0.7697	0.7378	0.7466	0.7693	0.7717	0.7732	0.7985	0.7896	0.7877	0.7987	0.7737	0.7655	0.7596	0.7453	0.7514
United Kingdom	0.8912	0.8866	0.9119	0.9088	0.9562	0.9573	0.9589	0.9548	0.9823	1	1	1	1	1	1	1	1	1	1	1

As mentioned above, we should take into consideration that DEA can measure “relative” efficiency, but not “absolute” efficiency. It compares an operating unit with a subset of peers and not with a theoretical maximum performance (Colbert et al. 2000). Thus, there is always a gap to improve in the real life. There is the possibility that coming years are more efficient than the previous; hence, the efficiency scores are expected to change. This further clarification is very important for the first hypothesis that the energy sector can improve its “sustainable” efficiency: the DEA method allows us to display that there is always the capability of improving our practices. Thus, the first hypothesis can be interpreted as correct. Once energy efficiency scores of the entire EU are obtained, the next step of the analysis is to estimate model (2), described in the “The independent variables and the modeling methodology” section.

### The independent variables and the modeling methodology

Next, after obtaining the energy efficiency sustainable index, the analysis explores whether the governance quality variables can explain this type of efficiency. Governance quality can be interpreted as the ability of the Government to ensure a framework for inhabitants of a country living justly. In the words of The Quality of Government (QoG) Institute, governance quality lies in trustworthy, reliable, impartial, uncorrupt, and competent government institutions.

The methodology used for obtaining the results is explained in the “Methodological analysis and final results” section.

Therefore, our primary model will be that of model (2), defined as follows:

2 $$ \mathbf{E}={\beta}_0+{\beta}_1\mathbf{I}\mathbf{1}+{\beta}_2\mathbf{I}\mathbf{2}+{\beta}_3\mathbf{I}\mathbf{3}+\mathbf{u}, $$

where variables are described below. Data are obtained from the World Bank public database^7^ and the Fraser Institute^8^, while they are all on an annual basis.

- **E**: Energy efficiency sustainable index.
- **I1**: Corruption. Taking the definition of the World Bank, “Control of Corruption captures perceptions of the extent to which public power is exercised for private gain, including both petty and grand forms of corruption, as well as “capture” of the state by elites and private interests.”
- **I2**: Regulatory quality. Taking the definition of the World Bank, “*Regulatory Quality captures perceptions of the ability of the government to formulate and implement sound policies and regulations that permit and promote private sector development*.”
- **I3**: Economic freedom index. The index is obtained from Fraser Institute and, according to the organization, it measures the degree of economic freedom present in five major areas: size of government, legal system and security of property rights, sound money, freedom to trade internationally, and corruption.
- **u**: the error term.
  The analysis spans the period 2002–2014, based on data availability.

**I1** and **I2** are two of the Worldwide Governance Indicators developed by the World Bank. Variable **I3**, developed by the Fraser Institute, is the general variable that measures what we have named as quality of Government. The rationale for introducing also **I1** and **I2** is the importance of these two variables in a country and for governance quality particularly (it is interesting to study their individual effect on **E**). The World Bank also developed four more indicators: government effectiveness, rule of law political stability and, voice and accountability. The first two we consider are measured by the three variables used; thus, if we introduce these two as well, we may add redundant information. The last two World Bank indicators reflect severe problems of democracy and freedom, that is not the case of the European Union.

Regarding the expected importance of each variable, we may anticipate that variables **I1** and **I2** could be the most relevant because they are more specific than **I3**, which includes more aspects of the economy that could not be influential for sustainable energy efficiency. In the “Empirical analysis” section, we will go deep into this fact and the results obtained.

## Methodological analysis and final results

Four panel unit root tests are considered: the inverse chi-squared test (*P*) proposed by Maddala and Wu (1999) and called the *P* test by Choi (2001), the modified *P* test (MP), the inverse normal test (IN), and the logit test (*L*), all three proposed by Choi (2001). Table 2 shows the panel unit root results. They clearly document that the variables **I1** and **I2** have a unit root in their levels and, thus, we consider their first differences for the next steps of the empirical analysis.

**Table 2:** Panel unit root test (*P* values)

Variable	*P*	MP	IN	*L*
**I1**	0.006	0.002	0.178	0.175
**I2**	0.012	0.006	0.258	0.212
**I3**	0	0	0	0

*P*, inverse chi-squared test; *MP*, modified *P*, test; *IN*, inverse normal test; *L*, logit test

**I1**, control of corruption; **I2**, regulatory quality; **I3**, Economic Freedom Index

Next, we need to choose the best methodology for our panel dataset; thus, the analysis runs three test hypotheses for both datasets:

- *F* test for individual and/or time effects: if we reject the null hypothesis, then the best option is to use the within model, if not, the pooling model.
- Lagrange Multiplier test for panel models (Breusch-Pagan test, Breusch and Pagan (1980)): if we reject the null hypothesis, then the best option is to use Generalized Least Squares (GLS), if not, the pooling model.
- Hausman test for panel models: if we reject the null hypothesis, then the best option is to use within model, if not, GLS.

In these three tests, the *P* values obtained are lower than 0.5, which indicates that the best method to use is Fixed Effects (the within model). The results are reported in Table 3.

**Table 3:** Panel data estimations of model (2) (fixed effects)

Variable	Estimations
_**I1**_	− 0.026 (− 0.771)
**I2**	0.044 (1.241)
**I3**	0.032^+^ (0.018)
*R*^2^	0.018
Adjusted *R*^2^	− 0.079
*F* statistic	1.772
*P* value (of *F* )	0.152
AIC	-
BIC	-

^+^Statistically significant at at 0.01 (99% level of confidence), . Values of the *t* statistics are presented in parentheses. **I1**, control of corruption; **I2**, regulatory quality; **I3**, Economic Freedom Index

The three explanatory variables are expected to have a positive impact on energy efficiency, which implies that higher levels of governance quality (measured as high control of corruption, quality of laws, or economic freedom) would infer higher efficiency of the energy sector and, hence, there would be higher levels of sustainable growth in the countries under consideration. However, the estimate results illustrate that the control of corruption carries a negative sign. This fact could be caused by a specific problem of the model: the presence of multicollinearity.

A priori, we could think that the institutional/government quality variables are highly collinear to each other; therefore, it is important to check the presence of collinearity in the study and, if it exists, to apply a specific methodology to deal with it. Although there have been some traditional methods applied in the presence of collinearity, such as ridge regression and partial least squares (PLS), these methods present some inconveniences and faults (Artigue and Smith (2019); Garćıa et al. (2015), (2017);

Salmerón et al. (2016a, 2016b)). There is a “novel” method called residualization (or also regression with orthogonal variables) to deal with collinearity. This methodology has been used in some previous research (Ambridge et al. 2012; Cohen-Goldberg 2012; Jaeger 2010; Jorgenson 2006; Jorgenson and Burns 2007; Jorgenson and Clark 2009; Kentor and Kick 2008; Kuperman et al. 2008, 2010; Lemhöfer et al. 2008); however, these works do not use the method as in here and they only orthogonalize one or a few variables as a function of only one variable that is usually not included in the original model. One of the first comprehensive explanations of the method and characteristics is provided by Salmerón et al. (2016a, 2016b). Residualization substitutes one, some, or all the variables with the residuals obtained from an auxiliary regression. Due to the properties of the OLS estimation, the estimated residuals of any regression by OLS are orthogonal to all the explanatory variables used in the analysis. Thus, if the auxiliary model is estimated by OLS, the estimated residuals represent the part of the dependent variable that has no relation with the explanatory variables used. Indeed, if we orthogonalize our explanatory variables in the model, each explanatory variable is completely independent in the model: the principle, ceteris paribus, is strictly fulfilled and there are no relationships between explanatory variables in the regression model; in that sense, the variables are totally independent to each other and potential multicollinearity problems are mitigated. It is worth noting the interpretative point of view of this method. The researcher chooses the “correct” variable(s) to be isolated: not all the variables may be “deconstructed” by deleting some of them, in a way that researchers must be careful with the choosing variable. Additionally, with the residualization method, we obtain the same estimated residuals for both the initial and the modified model, and provided that we are using the same explained variable, we obtain the same values for the global characteristics of the model (*F* statistic, *R*^2^, etc.). Moreover, since residualization uses OLS properties, hereinafter the analysis takes the mean of the period for each variable (cross-sectional data).

Before applying the residualization method, it is imperative to check out the presence of multicollinearity. One of the most widely applied measures to detect collinearity is the Variance Inflation Factor (VIF). The VIF, presented by Marquardt (1970), is usually taken the value of 10 as the frontier; any values higher than 10 detect collinearity problems. The VIFs for the variables, after calculating the mean for the period in all of them, are higher than 10 for one of the three explanatory variables (i.e., VIF_**I1**_ = 8*.*226, VIF_**I2**_ = 16*.*157, and VIF_**I3**_ = 5*.*008), so it is a sign of strong collinearity across all variables. The results of residualization, along with those of OLS, are reported in Table 4.

**Table 4:** Residualization and OLS estimation of model (2)

Variable	OLS	Residualization
Intercept	1.172 (0.937)	-0.160 (-0.177)
**I1**	0.022 (0.211)	0.164** (3.067)
**I2**	0.450 (1.448)	0.450 (1.548)
**I3**	-0.142 (-0.722)	0.090 (0.709)
*R*^2^	0.564	0.564
Adjusted *R*^2^	0.507	0.507
*F* statistic	9.912	9.912
*p*-value (of *F* )	*<* 2*.*2 × 10^*−*16^	*<* 2*.*2 × 10^*−*16^
AIC	− 18.961	− 18.961
BIC	− 12.482	− 12.482

**Statistically significant at 0.001 (99.9% level of confidence), at 0.01 (99% level of confidence), at 0.05 (95% level of confidence) and at 0.1 (90% level of confidence), respectively. Values of the *t* statistics are presented in parentheses.

**I1**, control of corruption; **I2**, regulatory quality; **I3**, Economic Freedom Index

The results highlight satisfactory values in relevance to collinearity problems. In particular, the VIF results are lower than 10 (i.e., VIF_**I1**_ = VIF_**I3**_ = 2*.*094, VIF_**I2**_ = 1*.*000). Regarding the expected signs, it would be logical that all governance quality coefficients have a positive sign, which is the case of these new results. This implies that increments in each explanatory variable would make the dependent variable (i.e., energy improvements or energy efficiency) higher. Finally, as a disadvantage of these results, we can see that the estimated parameters are not all individually significant, although we have a good model: we have an acceptable *R*^2^ taking into account that we are only studying the influence of institutional variables on energy efficiency and our model is globally significant. We conclude that our variables are important in explaining the energy efficiency sustainable index, because on an individual basis (by studying three different simple linear regressions), each one contributes with an acceptable *R*^2^ to the dependent variable (Table 5).

**Table 5:** Individual simple regressions for each explanatory variable

Variable	Estimated parameter	*R*^2^
**I1**	0.192*** (5.090)	0.509
**I2**	0.389*** (5.503)	0.548
**I3**	0.373** (3.589)	0.340

***, **Statistically significant at 0.001 (99.9% level of confidence), at 0.01 (99% level of confidence), at 0.05 (95% level of confidence), and at 0.1 (90% level of confidence), respectively. Values of the *t* statistics are presented in parentheses.

**I1**, control of corruption; **I2**, regulatory quality; **I3**, Economic Freedom Index

### Robustness check: alternative definitions of the independent variables

To check the robustness of our baseline findings, this part of the analysis changes two of the three explanatory variables in model (2) by considering alternative definitions. The analysis maintains the definition of the **I3** = Economic Freedom Index variable since, to the best of our knowledge, there is not any other alternative index available. The new variables, **I1** and **I2**, are obtained from The Quality of Government (QoG) Institute^9^, and they are defined as follows:

- **I1**: Public sector corruption Index. As the QoG Institute notes, the index is formed by taking the average of the point estimates from a Bayesian factor analysis model of the indicators for public sector bribery and embezzlement?
- **I2**: Impartial administration. As the QoG Institute notes, this variable overlaps with the concept of the rule of law; thus, it emphasizes the liberal aspects of democracy.
- **I3**: Economic freedom index.

It is important to note that our primary goal is to determine whether the characteristics of governments have any influence on energy efficiency, and hence, on the environmental degradation. As mentioned in the “Introduction” section, there are certain important government characteristics that could potentially affect environmental sustainability, such as the quality of political institutions or governance, well-defined property rights, and the role of the State or the Government (Magnani 2000; Rivera-Batiz 2002; Dinda 2004; Farzin and Bond 2006; Gnonlonfin et al. 2017).

The new dataset spans the period 2000 to 2014 and only 21 countries out of the EU-28. Table 6 includes only panel data estimations, considering the estimation by random effects. The findings indicate the presence of similar results as before. Furthermore, the estimates do not display any multicollinearity across the new variables. In terms of the GLS model, it is clearly documented that all of the governance quality variables are statistically significant at least at 10% in explaining energy efficiency scores. The findings illustrate the expected signs. Finally, as a disadvantage, the use of these new variables leads to a low *R*^2^, although the model is globally significant. This fact can be provoked by data unavailability and a smaller sample; thus, we conclude that the best definition of the independent variable comes from the first one. Overall, the second hypothesis can be interpreted as accepted, i.e., institutional or governance quality variables are important in explaining efficiency scores in the energy sector.

**Table 6:** Panel data estimations of model (2) with alternative variables. GLS model (random effects)

Variable	Estimations
Intercept	− 0.286 (− 1.386) 0.263^+^
**I1**	(1.893)
**I2**	0.860*** (5.413) 0.040^+^
**I3**	(1.790)
*R*^2^	0.105
Adjusted *R*^2^	0.096
*F* statistic	12.131
*P* value (of *F*)	< 2.2 × 10^−6^
AIC	-
BIC	-

***, ^+^Statistically significant at 0.001 (99.9% level of confidence), at 0.01 (99% level of confidence), at 0.05 (95% level of confidence), and at 0.1 (90% level of confidence), respectively. Values of the *t* statistics are presented in parentheses.

**I1**, Public Sector Corruption Index; **I2**, impartial administration; **I3**, Economic Freedom Index

## Empirical analysis

In terms of energy efficiency scores, the results indicated that the most energy efficient countries are Germany and Luxembourg, followed by the Netherlands, the UK, and Belgium. By contrast, the worst energy efficient country member is Bulgaria, followed by Slovak Republic, Lithuania, and Latvia. Therefore, it is obvious that there are two groups of countries in relevance to the efficiency of their energy sector, while there is a nexus between energy efficiency and income. In other words, while the most efficient countries are in the group of the countries with higher GDP per capita on average, the inefficient energy economies are in the group with smaller GDP per capita. With a lower GDP per capita, the country has less available resources to invest in new energy technologies and technologies environmentally friendly. The findings also indicated that the energy efficiency scores did not change dramatically through the time span under consideration (they took values between 0.6 and 0.7), but it could be appreciated that the most efficient years were those of 2007, 2008, and 2009, while the worst values were at the beginning of the time period. The controversial issue here is the most efficient years, because we do not have an increasing tendency regarding the efficiency scores: the results presented a raising trend until 2009, when the values started to decrease again. A potential explanation could be the economic (both financial and sovereign debt) crisis in Europe during those years; it is expected, that the end of the crisis will contribute to the increase of those efficiency scores again.

In terms of the role of governance quality variables, we have different results depending on the method applied, but the main estimation results (fixed effects for panel data and residualization for cross-sectional data) indicated that:

- With higher levels for the control of corruption, which captures the extent to which public power is exercised for private gains, it is likely that governments invest properly quantities in each sector, not only in those where the public has strong invested interests. Particularly, this fact is very important in the energy sector where with a stronger control of corruption there would be more investments on the research in new technologies and clean energies than in traditional ones (i.e., coal). The results just confirm this with a positive value of the estimated parameter in the residualization estimates (i.e., a higher control of corruption is beneficial for energy efficiency).
- Regulatory quality captures the ability of the government to formulate and implement sound policies and regulations that permit and promote private sector developments. This implies that a higher quality of such policies, are expected to lead to similar results as before, while the energy sector will turn out to be more efficient. Once again, the estimates just confirm this hypothesis through the positive value of the estimated parameter.
- Finally, the Economic Freedom Index measures the degree of economic freedom in a country. As this index is interpreted as the degree of democracy in a country, higher levels of the index are expected to improve the efficiency in the energy sector. The estimates also confirm this hypothesis through the positive value of the estimated parameter.

In terms of the importance of the independent variables used for explaining the sustainable or environmental energy efficiency scores, **E**, in the “Methodological analysis and final results” section, we have concluded that our variables are important in explaining the energy efficiency sustainable index, because each one contributes with an acceptable *R*^2^ to the dependent variable (Table 5). Furthermore, in the “The independent variables and the modeling methodology” section, we have anticipated that variables **I1** and **I2** could be the most relevant because they are more specific than **I3**, which includes more aspects of the economy that could not be influential for sustainable energy efficiency. Table 5 shows that variables **I1** and **I2** are the most important in the study: the correspondent parameters are statistically significant with a confidence level of 99.9%, while the parameter of variable **I3** is statistically significant with a confidence level of 99%. This fact means the three variables are relevant, but **I1** and **I2** predict more proportion of the variance in the dependent variable, **E**, than **I3** (Table 6).

## Discussion and conclusion

The paper attempted to investigate the empirical role of certain institutional variables for energy efficiency across all members of the EU-28 (excluding Malta). Overall, the empirical evidence indicated that the hypothesis that effective policies would have a positive effect on environmental conservation, especially for countries that exhibit carbon-emitting attitudes (Tajudeen et al. 2018), like the EU-28 group. With the principal goal of clarifying the role of governance quality variables in energy efficiency, we first obtained environmental energy efficiency scores across all members of the EU-28 (excluding Malta due to data unavailability) over the period 1995–2014. The use of environmental DEA, which employs not only desirable outputs but also undesirable ones to calculate the scores can be interpreted as an actual trend. We concluded from the efficiency scores that efficiency in the energy sector and income levels move together. Additionally, as it has been said in the “The dependent variable: the DEA and energy efficiency sustainable index” section, the DEA method allows us to display that there is always the capability of improving our practices. Thus, by using this method to obtain the energy efficiency scores, the first hypothesis can be interpreted as correct: there is a gap to improve the energy sector in terms of sustainable efficiency. Once the efficiency scores were obtained, the analysis estimated how governance quality impacted efficiency scores. The findings indicated that they played a key role in explaining the environmental energy efficiency. Hence, the second hypothesis of the work can be interpreted as correct as well: governance quality is important in explaining the sustainable efficiency of the energy sector.

The energy sector is a fundamental part of environmental policies, because the improvement on environment is generally due to the decrease of greenhouse gas (GHG) emissions. Lesser GHG emissions should come from a strong and innovative energy sector. As we have stated in the “Introduction” section, a large number of studies have already confirmed the bidirectional causality between energy sector and environmental policies, so the reader could think both the energy sector plays a pivotal role in environmental policies and environmental policies regulate energy sector. Anyway, with the presence of better institutions, investments in various economic sectors should be fair and appropriate. Particularly, in the case of the EU-28, that has a pro-active environmentalism and climate change is a challenge for this group of countries. Any improvements in governance quality are expected to positively impact on energy efficiency. However, the big question that arises is: how these countries might improve their practices to that end? When some governments pass a new law across all sectors in the economy (i.e., environment, energy, education, health, economy, and democracy), the first objective is to test whether the actions associated with it have any practical implications. In line with this, it is worthwhile to delve into the rebound effect. As Greening et al. (2000) (and references therein) said, “the term was first applied narrowly to the direct increase in demand for an energy service whose supply had increased as a result of improvements in technical efficiency in the use of energy.” In future research, this rebound or “take-back” effect will be interesting to study as well. Apart from this effect, a handy question would be to ask whether the law is efficient, taking into account all relevant variables and other policies that affect the new one. Moreover, we need to explore whether it has changed something, or the new practices have maintained the country as the starting point. It is clear that we have studied the EU-28 which is a group of developed countries with strong levels of democracy, while they have to fulfill some tight and good practices to remain within, so the controversial issue here is that countries must developed practical and useful actions, and test if they achieve the expected results. All the previous means that the first step when a new practice is implemented by the Government has to be to increment the “power” of it, does it work? If it does, quality of governments will be higher and perceptions of inhabitants and “neighbors” about the country, in general, and Government, in particular, will be better. This is essential in the way to make a better world, not only for energy sector or environmental issues but also for other aspects of the economy, like education in example.

## Footnotes

1 https://es.greenpeace.org/es/trabajamos-en/cambio-climatico/

2 https://climate.nasa.gov

3 https://europa.eu/european-union/topics/

4 https://europa.eu/european-union/topics/energy_en

5 https://europa.eu/european-union/topics/

6 Our data are available in the World Bank website (http://databank.worldbank.org/data/source/world-development-indicators).

7 http://databank.worldbank.org/data/databases.aspx

8 https://www.fraserinstitute.org/economic-freedom/dataset?geozone=world&year=2015&min-year=2&max-year=0&filter=0&sort-field=regulation&sort-reversed=1&page=dataset

9 https://qog.pol.gu.se/data

## References

[CR1] Ahmad A, Zhao Y, Shahbaz M, Bano S, Zhang Z, Wang S, Liu Y (2016). Carbon emissions, energy consumption and economic growth: an aggregate and disaggregate analysis of the Indian economy. Energy Policy.

[CR2] Aklin M, Bayer P, Harish S, Urpelainen J (2013). Understanding environmental policy preferences: new evidence from Brazil. Ecol Econ.

[CR3] Ambridge B, Pine J, Rowland C (2012). Semantics versus statistics in the retreat from locative overgeneralization errors. Cognition.

[CR4] Anderson W, Mizak D (2006). Politics of environmental law: political ideology, elitism or urban-rural interests?. Public Choice.

[CR5] Apergis N, Cooray A (2017). Economic freedom and income inequality: evidence from a panel of global economies? A Linear and a Non-Linear Long- Run Analysis. Manch Sch.

[CR6] Apergis N, Payne J (2014). Renewable energy, output, CO_2_ emissions, and fossil fuel prices in Central America: evidence from a nonlinear panel smooth transition vector error correction model. Energy Econ.

[CR7] Apergis N, Payne J, Menyah K, Wolde-Rufael Y (2010). On the causal dynamics between emissions, nuclear energy, renewable energy, and economic growth. Ecol Econ.

[CR8] Apergis N, Dincer O, Payne J (2014). Economic freedom and income inequality revisited: evidence from a panel error correction model. Contemp Econ Policy.

[CR9] Apergis N, Jebli M, Youssef S (2018). Does renewable energy consumption and health expenditures decrease carbon dioxide emissions? Evidence for sub-Saharan Africa countries. Renew Energy.

[CR10] Artigue H, Smith G (2019). The principal problem with principal components regression. Cogent Mathematics & Statistics.

[CR11] Balk B, De Koster M, Kaps C, Zosé J (2017) An evaluation of cross-efficiency methods, applied to measuring warehouse performance. Working paper (draft, December 2017)

[CR12] Bano S, Zhao Y, Ahmad A, Wang S, Liu Y (2018). Identifying the impacts of human capital on carbon emissions in Pakistan. Journal of Cleaner Production.

[CR13] Barbier E (1997). Introduction to the Environmental Kuznets Curve special issue. Environ Dev Econ.

[CR14] Barros C, Gil-Alana L, Payne J (2013). US disaggregated renewable energy consumption: persistence and long memory behavior. Energy Econ.

[CR15] Begum R, Sohag K, Abdullah S, Jaafar M (2015). CO_2_ emissions, energy con- sumption, economic and population growth in Malaysia. Renew Sust Energ Rev.

[CR16] Berggren N (2003). The benefits of economic freedom: a survey. The independent review.

[CR17] Bernauer T, Koubi V (2009). Effects of political institutions on air quality. Ecol Econ.

[CR18] Bhattarai M, Hammig M (2001). Institutions and the Environmental Kuznets Curve for deforestation: a crosscountry analysis for Latin America, Africa and Asia. World Dev.

[CR19] Bhattarai M, Hammig M (2004). Governance, economic policy, and the en- vironmental Kuznets curve for natural tropical forests. Environ Dev Econ.

[CR20] Blühdorn I, Welsh I (2007). Eco-politics beyond the paradigm of sustainability: a conceptual framework and research agenda. Environmental politics.

[CR21] Breusch T, Pagan A (1980). The Lagrange Multiplier test and its applications to model specification in econometrics. Rev Econ Stud.

[CR22] Cecchini L, Venanzi S, Pierri A, Chiorri M (2018). Environmental efficiency analysis and estimation of CO_2_ abatement costs in dairy cattle farms in Umbria (Italy): a SBM-DEA model with undesirable output. J Clean Prod.

[CR23] Charnes A, Cooper W, Rhodes E (1978). Measuring the efficiency of decision making units. Eur J Oper Res.

[CR24] Choi I (2001). Unit root tests for panel data. J Int Money Financ.

[CR25] Chortareas G, Girardone C, Ventouri A (2011). Financial frictions, bank efficiency and risk: evidence from the Eurozone. J Bus Financ Acc.

[CR26] Chortareas G, Girardone C, Ventouri A (2012). Bank supervision, regulation, and efficiency: evidence from the European Union. J Financ Stab.

[CR27] Chortareas G, Girardone C, Ventouri A (2013). Financial freedom and bank efficiency: evidence from the European Union. J Bank Financ.

[CR28] Chortareas G, Kapetanios G, Ventouri A (2016). Credit market freedom and cost efficiency in US state banking. J Empir Financ.

[CR29] Cohen-Goldberg A (2012). Phonological competition within the word: evidence from the phoneme similarity effect in spoken production. J Mem Lang.

[CR30] Colbert A, Levary R, Shaner M (2000). Determining the relative efficiency of MBA programs using DEA. Eur J Oper Res.

[CR31] Coondoo D, Dinda S (2002). Causality between income and emission: a country group-specific econometric analysis. Ecol Econ.

[CR32] De Haan J, Lundström S, Sturm J (2006). Market-oriented institutions and policies and economic growth: a critical survey. Journal of Economic Surveys.

[CR33] Deacon Robert T. (1994). Deforestation and the Rule of Law in a Cross-Section of Countries. Land Economics.

[CR34] Demirgüç-Kunt A, Laeven L, Levine R (2003) Regulations, market structure, institutions, and the cost of financial intermediation. Tech. rep., National Bureau of Economic Research

[CR35] Dinda S (2004). Environmental Kuznets curve hypothesis: a survey. Ecol Econ.

[CR36] Eastin J, Prakash A (2013). Economic development and gender equality: is there a gender Kuznets curve?. World Polit.

[CR37] Färe R, Grosskopf S, Lovell C, Pasurka C (1989). Multilateral productivity comparisons when some outputs are undesirable: a nonparametric approach. Rev Econ Stat.

[CR38] Farzin Y, Bond C (2006). Democracy and environmental quality. J Dev Econ.

[CR39] Garćıa CB, Garćıa J, López-Martín MM, Salmerón R (2015). Collinearity: revisiting the variance inflation factor in ridge regression. J Appl Stat.

[CR40] Garćıa C, Garćıa CB, Salmeró R, Garćıa J (2017). Regresi’on con variables ortogonales y regresi’on alzada en el modelo STIRPAT. Estudios de Economa Aplicada.

[CR41] Gnonlonfin A, Kocoglu Y, Peridy N (2017). Municipal solid waste and development: the Environmental Kuznets Curve evidence for Mediterranean countries. Region et Developpement.

[CR42] Greening L, Greene D, Difiglio C (2000). Energy efficiency and consumption - the rebound effect - a survey. Energy Policy.

[CR43] Gwartney J, Holcombe R, Lawson R (2006). Institutions and the impact of investment on growth. Kyklos.

[CR44] Jaeger T (2010). Redundancy and reduction: speakers manage syntactic information density. Cogn Psychol.

[CR45] Jebli M, Youssef S, Ozturk I (2016). Testing Environmental Kuznets Curve hypothesis: the role of renewable and non-renewable energy consumption and trade in OECD countries. Ecol Indic.

[CR46] Jorgenson A (2006). Global warming and the neglected greenhouse gas: A cross-national study of the social causes of methane emissions intensity, 1995. Social Forces.

[CR47] Jorgenson A, Burns T (2007). The political-economic causes of change in the ecological footprints of nations, 1991-2001: a quantitative investigation. Social Science Research.

[CR48] Jorgenson A, Clark B (2009). The economy, military, and ecologically unequal exchange relationships in comparative perspective: a panel study of the eco- logical footprints of nations, 1975-2000. Soc Probl.

[CR49] Kentor J, Kick E (2008). Bringing the military back in: military expenditures and economic growth 1990 to 2003. Journal of World-Systems Research.

[CR50] Komal R, Abbas F (2015). Linking financial development, economic growth and energy consumption in Pakistan. Renew Sust Energ Rev.

[CR51] Kuperman V, Bertram R, Baayen R (2008). Morphological dynamics in compound processing. Lang Cogn Process.

[CR52] Kuperman V, Bertram R, Baayen R (2010). Processing trade-offs in the reading of Dutch derived words. J Mem Lang.

[CR53] Lemhöfer K, Dijkstra T, Schriefers H, Baayen R, Grainger J, Zwitserlood P (2008). Native language influences on word recognition in a second language: a megastudy. J Exp Psychol Learn Mem Cogn.

[CR54] Li H, Squire L, Zou H (1998). Explaining international and intertemporal variations in income inequality. Econ J.

[CR55] Lin K, Doan A, Doong S (2016). Changes in ownership structure and bank efficiency in Asian developing countries: the role of financial freedom. International Review of Economics & Finance.

[CR56] Liobikieǹe G, Butkus M (2018). The challenges and opportunities of climate change policy under different stages of economic development. Sci Total Environ.

[CR57] Lloyd P (2017). The role of energy in development. Journal of Energy in Southern Africa.

[CR58] Maddala G, Wu S (1999). A Comparative study of unit root tests with panel data and a new simple test. Oxf Bull Econ Stat.

[CR59] Magnani E (2000). The Environmental Kuznets Curve, environmental protection policy and income distribution. Ecol Econ.

[CR60] Marquardt D (1970). Generalized inverses, ridge regression, biased linear estimation and nonlinear estimation. Technometrics.

[CR61] Mikayilov J, Galeotti M, Hasanov F (2018). The Impact of Economic Growth on CO2 Emissions in Azerbaijan. J Clean Prod.

[CR62] Moutinho V, Madaleno M, Robaina M (2017). The economic and environmental efficiency assessment in EU cross-country: evidence from DEA and quantile regression approach. Ecol Indic.

[CR63] Nasreen S, Anwar S (2014). Causal relationship between trade openness, eco- nomic growth and energy consumption: a panel data analysis of Asian coun- tries. Energy Policy.

[CR64] Norton S (1998) Property rights, the environment, and economic well-being. Who Owns the Environment pp 37–54, oxford, England: Rowan and Little- field

[CR65] Omer A (2008). Energy, environment and sustainable development. Renew Sust Energ Rev.

[CR66] Pitlik H (2002). The path of liberalization and economic growth. Kyklos.

[CR67] Rivera-Batiz F (2002). Democracy, governance, and economic growth: theory and evidence. Rev Dev Econ.

[CR68] Robaina-Alves M, Moutinho V, Macedo P (2015). A new frontier approach to model the eco-efficiency in European countries. J Clean Prod.

[CR69] Sadorsky P (2009). Renewable energy consumption and income in emerging economies. Energy Policy.

[CR70] Sadorsky P (2009). Renewable energy consumption, CO_2_ emissions and oil prices in the G7 countries. Energy Econ.

[CR71] Salahodjaev R (2016). Does intelligence improve environmental sustainability? An empirical test. Sustain Dev.

[CR72] Salmerón R, Garćıa J, Garćıa CB, Garćıa C (2016). Treatment of collinearity through orthogonal regression: an economic application. Bolet’ın de Es- tad́ıstica e Investigaci’on Operativa.

[CR73] Salmerón R, Garćıa J, López-Martín MM, Garćıa CB (2016). Collinearity diagnostic applied in ridge estimation through the variance inflation factor. J Appl Stat.

[CR74] Samuelson P (1976). Economics of forestry in an evolving society. Econ Inq.

[CR75] Scheel H (2001). Undesirable outputs in efficiency valuations. Eur J Oper Res.

[CR76] Seiford L, Zhu J (2002). Modeling undesirable factors in efficiency evaluation. Eur J Oper Res.

[CR77] Shahbaz M, Ozturk I, Afza T, Ali A (2013). Revisiting the environmental Kuznets curve in a global economy. Renew Sust Energ Rev.

[CR78] Shahbaz M, Shahzad S, Alam S, Apergis N (2018) Globalisation, economic growth and energy consumption in the BRICS region: the importance of asymmetries. The Journal of International Trade & Economic Development pp 1–25

[CR79] Sohag K, Begum R, Abdullah S, Jaafar M (2015). Dynamics of energy use, technological innovation, economic growth and trade openness in Malaysia. Energy.

[CR80] Tajudeen I, Wossink A, Banerjee P (2018). How significant is energy efficiency to mitigate CO_2_ emissions? Evidence from OECD countries. Energy Econ.

[CR81] Tronchin L, Manfren M, Nastasi B (2018). Energy efficiency, demand side man- agement and energy storage technologies - a critical analysis of possible paths of integration in the built environment. Renew Sust Energ Rev.

[CR82] Tugcu C, Ozturk I, Aslan A (2012). Renewable and non-renewable energy consumption and economic growth relationship revisited: evidence from G7 countries. Energy Econ.

[CR83] Vencheh A, Matin R, Kajani M (2005). Undesirable factors in efficiency mea- surement. Appl Math Comput.

[CR84] Wang Q, Chiu Y, Chiu C (2015). Driving factors behind carbon dioxide emissions in China: A modified production-theoretical decomposition analysis. Energy Econ.

[CR85] Wang S, Li Q, Fang C, Zhou C (2016). The relationship between economic growth, energy consumption, and CO2 emissions: empirical evidence from China. Sci Total Environ.

[CR86] Wang Y, Chen L, Kubota J (2016). The relationship between urbanization, energy use and carbon emissions: evidence from a panel of Association of Southeast Asian Nations (ASEAN) countries. J Clean Prod.

[CR87] Zhou P, Ang B (2008). Linear programming models for measuring economy-wide energy efficiency performance. Energy Policy.

